# Investigation of a method to estimate the average speed of sound using phase variances of element signals for ultrasound compound imaging

**DOI:** 10.1007/s10396-023-01378-9

**Published:** 2023-11-10

**Authors:** Ryo Nagaoka, Masaaki Omura, Hideyuki Hasegawa

**Affiliations:** Faculty of Engineering, University of Toyama, 3190 Gofuku, Toyama, 930-8555 Japan

**Keywords:** Average speed of sound, Coherence factor, Phase variance, Ultrasound compound imaging

## Abstract

**Purpose:**

In the receive beamforming of an ultrasonography system, a B-mode image is reconstructed by assuming an average speed of sound (SoS) as a constant value. In our previous studies, we proposed a method for estimating the average SoS based on the coherence factor (CF) and the reciprocal of phase variances of element signals in delay-and-sum (DAS) beamforming. In this paper, we investigate the accuracy of estimation of the average SoS for compound imaging.

**Methods:**

For this purpose, two numerical simulations were performed with k-Wave software. Also, the estimation methods based on the CF and the reciprocal were applied to in vivo data from the common carotid artery, and B-mode images were reconstructed using the estimated average SoS.

**Results:**

In the first numerical simulation using an inhomogeneous phantom, the relationship between the accuracy and the transmission angles for the estimation was investigated, and the root mean squared errors (RMSEs) of estimates obtained based on the CF and the reciprocal of the phase variance were 1.25 ± 0.09, and 0.765 ± 0.17% at the transmission sequence of steering angles of (− 10°, − 5°, 0°, 5°, 10°), respectively. In the second numerical simulation using a cyst phantom, lateral resolutions were improved by reconstructing the image using the estimates obtained using the proposed strategy (reciprocal). By the proposed strategy, improvement of the continuity of the lumen–intima interface in the lateral direction was observed in the in vivo experiment.

**Conclusion:**

Consequently, the results indicated that the proposed strategy was beneficial for estimation of the average SoS and image reconstruction.

## Introduction

In an ultrasonography system, an ultrasonic B-mode image is reconstructed by assuming an average speed of sound (SoS) as a constant value of 1530 or 1540 m/s. However, the SoS is dependent on the type of tissue, such as skin, muscle, lipid, and bone, and the SoS values even in the same kind of tissue differ. The mismatch between the assumed and actual average SoS in a beamforming process results in degradation of the reconstructed images. Note that the average SoS is an average value of the SoS on a path from an ultrasonic element to an interest point. Many studies have been conducted to solve this issue from the viewpoints of correction of phase aberration [1–3] and estimation of the average (or local) SoS. The phase aberration due to variations of the SoS can be estimated using cross-correlation of received signals to correct the effects based on the estimates. In this paper, we focus on methods for estimating the average SoS.

Some research groups have proposed a method for estimating the SoS using echo signals from scatterers [4, 5] and crossed ultrasonic beams [6]. Cho et al. have proposed a method of estimating the SoS by comparing theoretical delay times of signals received by transducer elements [7]. Abe et al. have proposed an estimation method that compares the measured and theoretical delay times in a region of interest (ROI) divided into several regions [8, 9]. Monjazebi et al. have proposed a method based on a similar principle for synthetic aperture imaging [10]. Imbault et al. and Nitta et al. have proposed an SoS estimation method based on the coherence of the channel signals [11–13]. Nitta et al. have also investigated relationships between the statistical properties of a distribution of the local SoS and average SoS by performing numerical simulation and phantom experiments [12]. They have also evaluated the relationship between longitudinal and shear wave speeds of the phantoms. The longitudinal wave speed is referred to as SoS in this paper [13]. Shin et al. have proposed an estimation method using non-blind deconvolution with a point spread function (PSF) obtained at different SoS [14]. The method proposed by Krücker et al. has estimated the SoS by evaluating geometric distortions of the images obtained at different transmission angles compared to a reference image [15]. In ultrasound computed tomography (USCT), the distribution of the local SoS can be estimated from the time-of-flight of the pulse wave transmitted from an emitter to a receiver by assuming that the transmitted pulse propagates along a straight path without refraction and diffraction [16, 17]. Jaeger et al. have proposed an estimation method using a linear array probe based on the principle of USCT [18–21]. First, plane waves at different angles were transmitted to tissues, and spherical waves backscattered from a point of interest (POI) were received using the full aperture of the probe. Then, receive beamforming was applied to the received signals to reconstruct an A-line signal at each transmitted angle. In terms of the A-line signals, although the propagating time of the spherical wave backscattered from the POI to the probe was independent of the transmitted angles, the propagation time of the plane wave transmitted from the probe to the POI was dependent on the angles. In their proposed method, the relationship between the propagation time and the transmitted angle was used for estimation of the SoS. Under the assumption of muscle as an anisotropic medium, Martiartu et al. investigated anisotropy of the SoS using a plane reflector [22].

Several research groups have proposed a method for estimating SoS based on evaluation indices of focusing qualities, such as phase [23–25], reflection matrix [26], coherence factor (CF) [27–32], and signal-to-noise ratio (SNR) [28]. In these estimation methods with phase, CF, and SNR, the evaluation index in the ROI was calculated under different assumed average SoSs, and then the average SoS, which maximizes or minimizes the index value, was determined as the estimate. In the referenced paper [31], local SoS were estimated from the average SoS under the assumption that a measurement target consisted of layered structures. Our research group has proposed an estimation method of the average SoS based on the phase, CF [33, 34], and SNR. In our previous research [30], a method based on the CF for ultrasound compound imaging [35] was verified by performing numerical simulations using k-Wave software [36, 37] to reveal a relationship between the estimation accuracy and transmission angles of plane waves. Also, while Yoon et al. have proposed an estimation method that minimizes variances of the phases of the delay-compensated element signals in DAS beamforming [23], the estimates have been obtained by maximizing a reciprocal of the variance of phases, similar to phase coherence factor (PCF) [38] in our previous studies [24, 25]. The results of these investigations indicated that the effects of signals from tissue boundaries on estimation of the SoS could be more suppressed using the proposed strategy. In the present study, we investigated the estimation accuracy of the method based on the reciprocal of the phase variance compared to that based on the CF. For this purpose, first, numerical simulations with the k-Wave software were performed to investigate the relationship between the estimation accuracy and transmission angles. Also, we compared the results with those obtained using the method based on the CF. Next, the effects of the signals from the tissue boundaries and degree of improvement of image quality using the estimates for image reconstruction were evaluated by performing numerical simulations using a cyst phantom containing wire targets. Finally, the estimation methods using both strategies were applied to in vivo data from a common carotid artery.

## Materials and methods

### Estimation method for average speed of sound

Let us define a complex delay-compensated signal received by the $i$-th ultrasonic element located at the position $x_{i}$ in the $x$ direction as $a\left( {x,z, i,\theta_{j} ,c_{a} } \right)$. The process of delay-compensation was performed using the following equations under the assumption that signals were scattered from a point scatterer [35].

1 $$a\left( {x,z, i,\theta_{j} ,c_{a} } \right) = {\text{rf}}\left( {t_{t} + t_{b} , i,\theta_{j} ,c_{a} } \right),$$

2 $$t_{t} = \frac{{z\cos \theta_{j} + x\sin \theta_{j} }}{{c_{a} }}$$

3 $$t_{b} = \frac{{\sqrt {\left( {x_{i} - x} \right)^{2} + z^{2} } }}{{c_{a} }},$$

where a variable ${\text{rf}}\left( {t, i,\theta_{j} ,c_{a} } \right)$ is the signal received by the $i$-th ultrasonic element at time $t$. Also, $t_{t}$ is a time of forward propagation of a plane wave with the $j$-th angle $\theta_{j}$, and $t_{b}$ is a time of backward propagation from a position $\left( {x, z} \right)$ of the point scatterer to the $i$-th ultrasonic element. The variable $c_{a}$ is an average SoS for the receive beamforming and estimation. Also, let us define a real part of the complex signal as $r\left( {x,z, i,\theta_{j} ,c_{a} } \right)$. The CF for estimation of the average SoS was calculated as

4 $${\text{CF}}\left( {x, z, \theta_{j} ,c_{a} } \right) = \frac{{\left| {\sum\limits_{i = 0}^{N - 1} {r\left( {x,z, i,\theta_{j} ,c_{a} } \right)} } \right|^{2} }}{{N\sum\limits_{i = 0}^{N - 1} {\left| {r\left( {x,z, i,\theta_{j} ,c_{a} } \right)} \right|}^{2} }},$$

where $N$ is the total number of active ultrasonic elements for beamforming [33, 34]. Then, the CFs in terms of all the transmission angles $\theta_{j}$ at each assumed average SoS were averaged with weighting values as

5 $${\text{CF}}_{{{\text{ave}}}} \left( {x, z, c_{a} } \right) = \mathop \sum \limits_{j = 1}^{M} w\left( {x, z, \theta_{j} ,c_{a} } \right) \cdot {\text{CF}}\left( {x, z, \theta_{j} ,c_{a} } \right),$$

where $M$ is the total number of plane wave transmissions for the estimation. Also, $w\left( {x, z, \theta_{j} ,c_{a} } \right)$ denotes the weighting values determined by the number of transmitted plane waves passing through a position $\left( {x, z} \right)$ and the weighting values were normalized by the transmission number to satisfy an equation of $\mathop \sum \limits_{j = 1}^{M} w\left( {x, z, \theta_{j} ,c_{a} } \right) = 1$. Next, a procedure for calculation of the reciprocal of the phase variance is described. The phase variance was calculated as

6 $${\text{Var}}_{{\text{p}}} \left( {x, z, \theta_{j} ,c_{a} } \right) = \frac{1}{N}\left[ {\mathop \sum \limits_{i = 0}^{N - 1} \left\{ {\angle a\left( {x,z, i,\theta_{j} ,c_{a} } \right) - \mu_{{\text{p}}} \left( {x,z, \theta_{j} ,c_{a} } \right)} \right\}^{2} } \right],$$

7 $$\mu_{{\text{p}}} \left( {x, z, \theta_{j} ,c_{a} } \right) = \frac{1}{N}\mathop \sum \limits_{i = 0}^{N - 1} \angle a\left( {x,z, i,\theta_{j} ,c_{a} } \right),$$

where $\angle *$ denotes an angle of a complex value, and $\mu_{{\text{p}}}$ is the average value of the phase of the complex delay-compensated signals in the beamforming. Then, the reciprocal of the phase variance for estimation of the average SoS was calculated as

8 $${\text{Var}}_{{{\text{rp}}}} \left( {x, z, \theta_{j} ,c_{a} } \right) = \frac{1}{{\left| {{\text{Var}}_{{\text{p}}} \left( {x, z, \theta_{j} ,c_{a} } \right) + \lambda } \right|}},$$

where $\lambda$ is a stabilization parameter to prevent the reciprocal ${\text{Var}}_{{{\text{rp}}}} \left( {x, z, \theta_{j} ,c_{a} } \right)$ from being an infinite value when a term of ${\text{Var}}_{{\text{p}}} \left( {x, z, \theta_{j} ,c_{a} } \right)$ in the denominator comes close to being zero. In this paper, the stabilization parameter was set to 1. In a manner similar to the average operation of the CF, the reciprocals of the phase variances in terms of all the transmission angles were averaged with the weighting values as

9 $${\text{Var}}_{{{\text{ave}}}} \left( {x, z, c_{a} } \right) = \mathop \sum \limits_{j = 1}^{M} w\left( {x, z, \theta_{j} ,c_{a} } \right) \cdot {\text{Var}}_{{{\text{rp}}}} \left( {x, z, \theta_{j} ,c_{a} } \right).$$

The average value of the CF, ${\text{CF}}_{{{\text{ave}}}} \left( {x_{ma} , z_{ma} , c_{a} } \right)$, or reciprocal ${\text{Var}}_{{{\text{ave}}}} \left( {x_{ma} , z_{ma} , c_{a} } \right)$ in a two-dimensional (2D) kernel was used to calculate an evaluation index ${\text{EI}}_{{{\text{CF}}}} \left( {x, z, c_{a} } \right)$ or ${\text{EI}}_{{{\text{Var}}}} \left( {x, z, c_{a} } \right)$ for estimation of the average SoS, respectively, as follows:

10 $${\text{EI}}_{{{\text{CF}}}} \left( {x, z, c_{a} } \right) = {\text{E}}_{R} \left[ {{\text{CF}}_{{{\text{ave}}}} \left( {x_{ma} , z_{ma} , c_{a} } \right)} \right],$$

11 $${\text{EI}}_{{{\text{Var}}}} \left( {x, z, c_{a} } \right) = {\text{E}}_{R} \left[ {{\text{Var}}_{{{\text{ave}}}} \left( {x_{ma} , z_{ma} , c_{a} } \right)} \right],$$

where ${\text{E}}_{R} \left[ {*} \right]$ denotes an average operation in the 2D kernel window $R$. In this average operation, a center position of the window was set as a position $\left( {x, z} \right)$. Also, the values of more than the average one at a position $\left( {x_{ma} , z_{ma} } \right)$ in the kernel were used for the average operation, which meant that only the values satisfying a relationship of ${\text{CF}}_{{{\text{ave}}}} \left( {x_{ma} , z_{ma} , c_{a} } \right) > {\text{E}}_{R} \left[ {{\text{CF}}_{{{\text{ave}}}} \left( {x, z, c_{a} } \right)} \right]$ or ${\text{Var}}_{{{\text{ave}}}} \left( {x_{ma} , z_{ma} , c_{a} } \right) > {\text{E}}_{R} \left[ {{\text{Var}}_{{{\text{ave}}}} \left( {x, z, c_{a} } \right)} \right]$ were used for the calculation, to suppress effects from signals with low SNR and noise. In this investigation, the kernel sizes in lateral $\left( x \right)$ and depth $\left( z \right)$ directions were empirically chosen as 2.00 mm and 2.46 mm, respectively. The distribution of the average SoS was obtained by performing the estimation at each position $\left( {x, z} \right)$ in the whole ROI. In this estimation, intervals in the lateral and depth directions were set to 0.2 mm and 0.0246 mm, respectively, which corresponded to one pixel in both directions. When changing the assumed average SoS $c_{a}$, the average SoS, which maximizes the evaluation index in Eq. (10) or (11), was determined as the estimated average SoS $\hat{c}_{e} \left( {x, z} \right)$. In the present study, the assumed average SoS was changed from 1450 m/s to 1700 m/s at an interval of 5 m/s.

### Numerical simulations

In this paper, two numerical simulations with inhomogeneous and cyst phantoms were conducted using the k-Wave software [36, 37]. Table 1 shows parameters for the numerical simulations. In this experiment, a linear array probe with a center frequency of 7.5 MHz was simulated. The element pitch was set to 0.2 mm and the number of elements was 192. A region for the simulation was divided with a spatial interval of 0.02 mm in both the depth and lateral directions, and the time step was 5.33 ns. Also, the Courant–Friedrichs–Lewy (CFL) value was 0.4107. Plane waves with an apodization function using a Tukey window with a coefficient of 0.2 were transmitted to the simulation phantoms to obtain element signals. The element signals obtained with the simulation were down-sampled from 187.5 MHz to 31.25 MHz. Hence, the sampling frequency was set to 31.25 MHz, which was identical to the sampling frequency used in the following in vivo experiments. The down-sampled element signals were used for estimation of the average SoS. Also, receive beamforming with a fixed F-number of 1 was applied to the identical signals.

**Table 1:** Parameters for numerical simulations

Properties of linear array probe			
Center frequency [MHz]		7.5	
Sampling frequency [MHz]		31.25	
Element pitch (kerf) [mm]		0.2 (0.02)	
The number of elements		192	
Mechanical properties of phantom			
Material	Sound speed [m/s]		Density [kg/m^3^]
Homogeneous medium	1540		1000
Inhomogeneous medium	1540 (SD: 1%)		1000 (SD: 1%)
Cyst region	1650 (SD: 1%)		1000 (SD: 1%)
Wire ($\phi$: 180 μm)	1800		1000
Grid size			
Spatial interval along axial direction [mm]		0.02	
Spatial interval along lateral direction [mm]		0.02	
Time step [ns]		5.33	
Value of Courant–Friedrichs–Lewy (CFL)		0.4107	

**Fig. 1:**
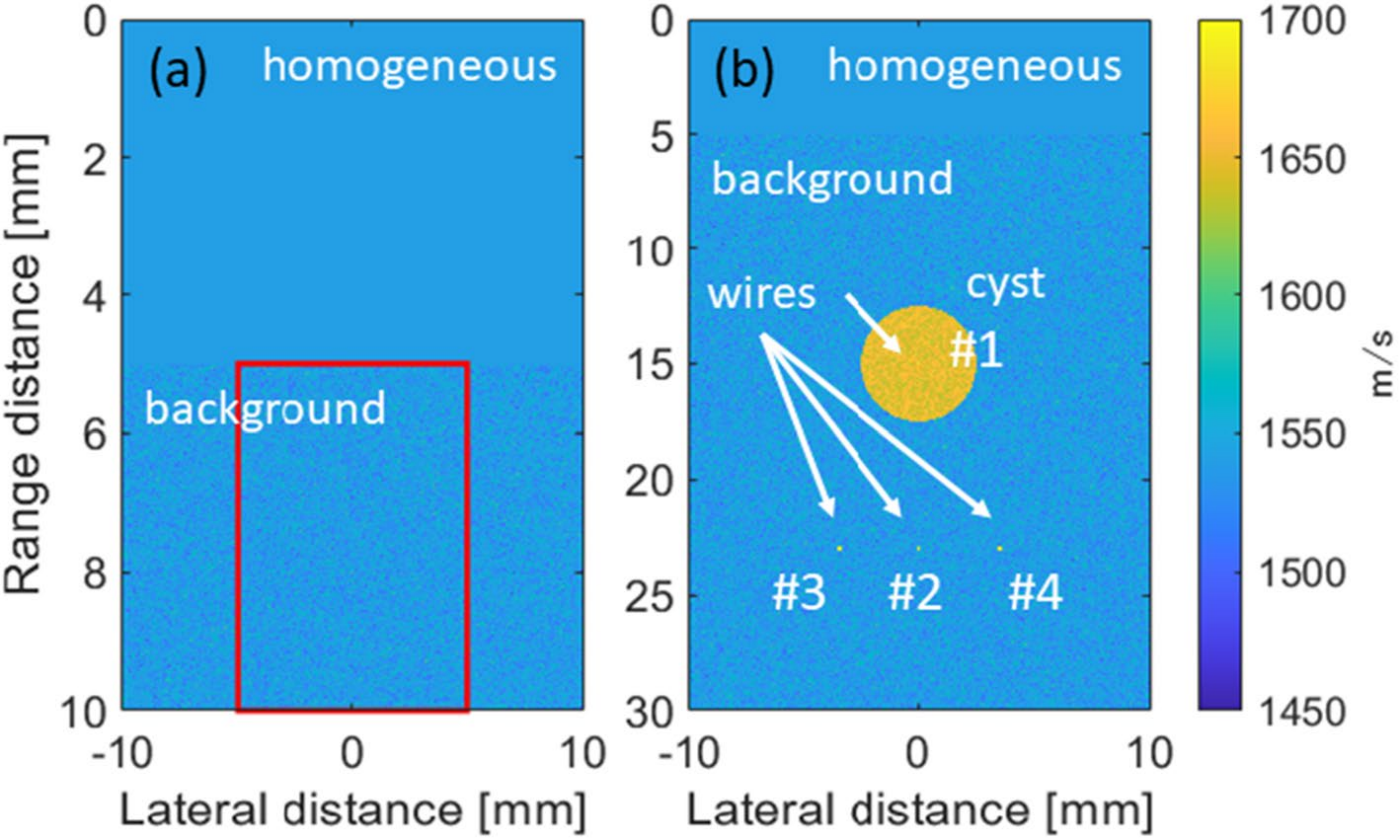
Distribution of assigned SoS in **a** inhomogeneous and **b** cyst phantom. Both the phantoms contained a uniform region with a SoS of 1540 m/s between the probe and phantom, referred to as the homogeneous region in this paper; Background region: 1540 m/s (SD: 1%), cyst region: 1650 m/s (SD: 1%)

In the numerical simulations using the inhomogeneous phantom shown in Fig. 1(a), the relationship between the angles of the transmitted plane wave $\theta_{j}$ and accuracy of the estimation method based on the two evaluation indices was investigated. The following seven transmission sequences of the steering angles were employed in the numerical simulations using the inhomogeneous phantom: (− 1°, − 0.5°, 0°, 0.5°, 1°), (− 3°, − 1.5°, 0°, 1.5°, 3°), (− 5°, − 2.5°, 0°, 2.5°, 5°), (− 7°, − 3.5°, 0°, 3.5°, 7°), (− 10°, − 5°, 0°, 5°, 10°), (− 12°, − 6°, 0°, 6°, 12°), and (− 15°, − 7.5°, 0°, 7.5°, 15°). The proper sequence of the steering angles chosen in this numerical simulation will be used in the successive numerical simulations and experiments. The average SoS of the inhomogeneous phantom was set to 1540 m/s, and its standard deviation (SD) was set to 1% of the average SoS (1540 m/s). The assigned SoS of the inhomogeneous phantom followed a normal distribution. Also, the distance from the surface of the ultrasonic array probe to the surface of the phantom was set to 5 mm. The SoS of this region, which was referred to as a homogeneous region, between the probe and phantom was set to a constant SoS of 1540 m/s. The estimation accuracy was evaluated based on absolute bias error (ABE) and root mean squared error (RMSE) in a region $R_{e}$ where the average SoS were estimated as

12 $${\text{ABE}} = \frac{{\left| {{\text{E}}_{{R_{e} }} \left[ {\hat{c}_{e} - c_{t} } \right]} \right|}}{{\left| {{\text{E}}_{{R_{e} }} \left[ {c_{t} \left( {x,z} \right)} \right]} \right|}} \times 100 = \frac{{\left| {{\text{E}}_{{R_{e} }} \left[ {\hat{c}_{e} - c_{t} } \right]} \right|}}{{\overline{c}_{t} }} \times 100 [\% ],$$

13 $${\text{RMSE}} = \frac{{\sqrt {{\text{E}}_{{R_{e} }} \left[ {\left( {\hat{c}_{e} - c_{t} } \right)^{2} } \right]} }}{{\left| {{\text{E}}_{{R_{e} }} \left[ {c_{t} \left( {x,z} \right)} \right]} \right|}} \times 100 = \frac{{\sqrt {{\text{E}}_{{R_{e} }} \left[ {\left( {\hat{c}_{e} - c_{t} } \right)^{2} } \right]} }}{{\overline{c}_{t} }} \times 100 [\% ],$$

where $c_{t} \left( {x,z} \right)$ and $\overline{c}_{t}$ are the assigned distribution of the true SoS and average SoS in the region $R_{e}$, respectively. As the 2D kernel window $R$ was sufficiently large in this study (2.00 mm $\times$ 2.46 mm in the lateral $\left( x \right)$ and depth $\left( z \right)$ directions), the denominator of Eqs. (12) and (13) corresponded to the mean value of the assigned SoS, i.e., 1540 m/s. This numerical simulation was performed three times to calculate the mean value and SD of the ABE and RMSE. Although an angle of 10° or 12° was proper for estimation based on the evaluate index ${\text{EI}}_{{{\text{CF}}}} \left( {x, z, c_{k} } \right)$ in our previous research [30], the number of numerical simulation trials was increased from one trial to three trials for comparison of the accuracy. In a trial, distributions of the SoS and density were calculated under the conditions described above, and the numerical simulation was performed using the calculated distributions for all the transmission sequences. Then, new distributions of the SoS and density were calculated for the next trial.

Next, numerical simulation using the cyst phantom shown in Fig. 1(b) was performed to investigate effects from the cyst region in terms of estimation accuracy and image quality. The effects from the angles for compounding the B-mode images were also evaluated in this numerical simulation. The mechanical properties of the background region corresponded to those of the inhomogeneous phantom described above. The assigned SoS of the background region also followed the normal distribution. The center position of the cyst region with a radius of 2.5 mm was set to a position $\left( {x, z} \right)$ of $\left( {0 {\text{ mm}}, 15 {\text{ mm}}} \right)$. Although the assigned SoS of the cyst region followed the normal distribution, SoS values greater than 1732.5 m/s (mean value + one standard deviation) and less than 1567.5 m/s (mean value − one standard deviation) were assigned as 1732.5 m/s and 1567.5 m/s, respectively. Also, the phantom contained four wires, indicated by arrows in Fig. 1(b). One wire was located at the center of the cyst region corresponding to a depth of 15 mm ($\# 1$), and the other ones were located at a depth of 23 mm ($\# 2$-$\# 4$). The homogeneous region with a SoS of 1540 m/s was located at a depth of 5 mm from the surfaces of the probe under the condition similar to the inhomogeneous phantom experiment. In this phantom experiment, a lateral spatial resolution, which was defined as a full width at half-maximum, obtained with respect to each wire in the B-mode image was evaluated. Also, the lateral resolutions ${\text{slr}}_{{{\text{CF}}}}$ and ${\text{slr}}_{{{\text{rec}}}}$ in the B-mode images reconstructed using the average SoS estimated based on the CF and reciprocal of the phase variance were compared with the resolution ${\text{slr}}_{{{\text{DAS}}}}$ reconstructed using a constant average SoS of 1540 m/s. Using the estimated average SoS in the image reconstruction (beamforming) process is referred to as feedback in this paper.

### In vivo experiments

In the in vivo experiment, RF echo signals from the common carotid artery of a healthy 49-year-old male were measured using a 7.5-MHz linear array probe (UST-5412, Fujifilm) connected to an ultrasonic programable measurement system (RSYS-0011; Microsonic Co., Ltd.) at a sampling frequency of 31.25 MHz. The number of ultrasonic elements in the linear array probe was 192. The most proper transmission sequence of the steering angles determined based on the results of the numerical simulations was used. Other parameters for image reconstruction and estimation were identical to those used in the numerical simulations. This study was approved by the institutional ethical committee and performed with the informed consent of the subject.

## Results

### Numerical simulations

**Fig. 2:**
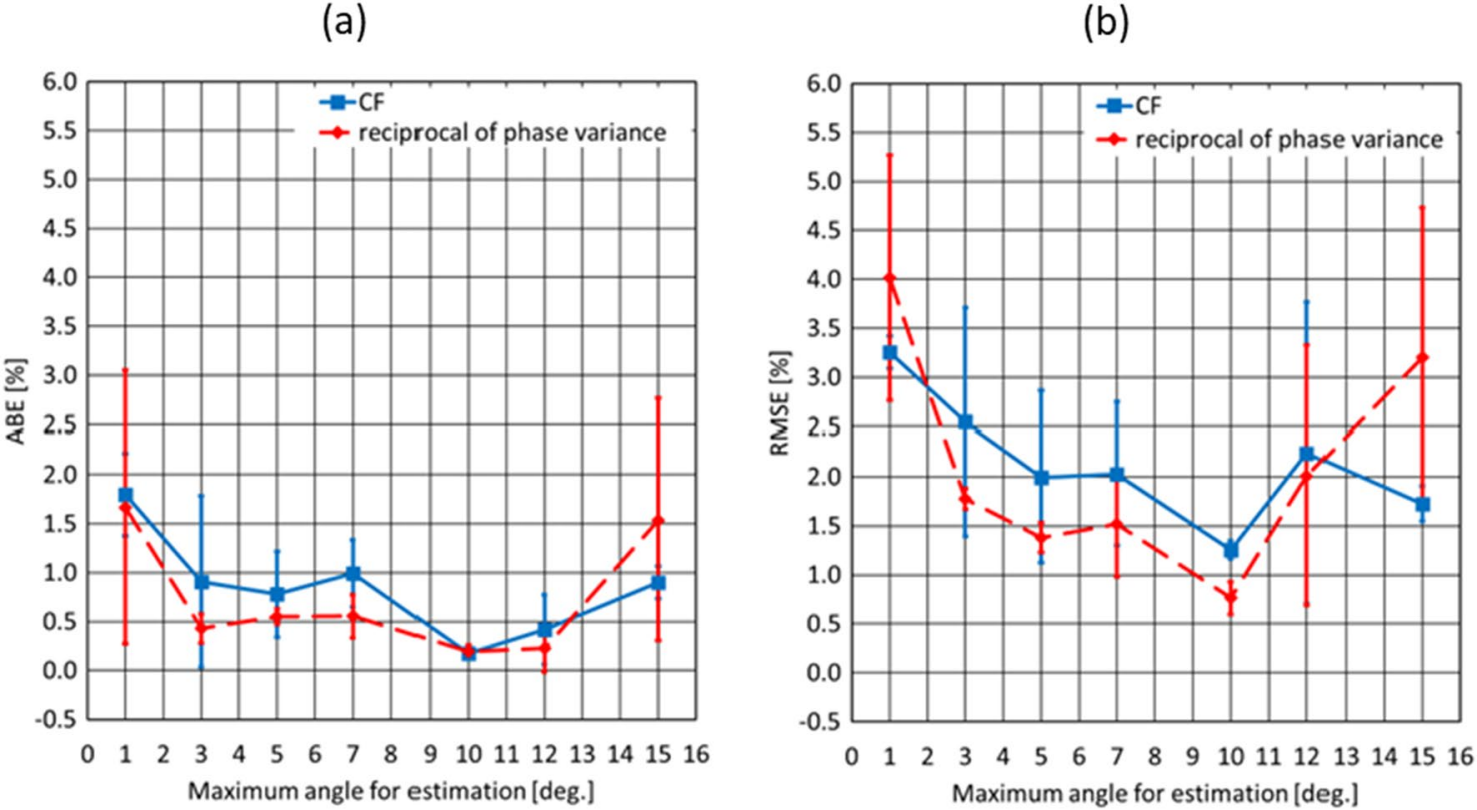
**a** ABE and **b** RMSE calculated in the ROI surrounded by a red square in Fig. 1(a) based on the CF and reciprocal of the phase variance under different maximum angles of the plane wave transmissions

Figure 2(a) and (b) shows the ABE and RMSE calculated from the results of the three trials in the ROI surrounded by the red square in Fig. 1(a) under the different maximum angles in the plane wave transmissions, respectively. The size of the ROI was 10 × 5 mm^2^ in the lateral and depth (range) directions. In Fig. 2(a) and (b), the error bars correspond to the SD values of the ABE and RMSE. As shown in Fig. 2(a) and (b), both the ABE and RMSE were the most suppressed at the transmission sequence of steering angles of (− 10°, − 5°, 0°, 5°, 10°). In this sequence, the RMSE of the estimates based on the CF and reciprocal of the phase variance were 1.25 ± 0.09 and 0.765 ± 0.17, respectively. As a result, the RMSE obtained using the reciprocal was slightly less than that obtained using the CF. It’s considered that this was because the ROI was adjacent to the boundary between the homogeneous and background regions and the estimates were affected by the boundary. In the following experiments using the cyst phantom, the transmission sequence of (− 10°, − 5°, 0°, 5°, 10°) was employed. Also, additional transmission sequences of (− 5°, − 2.5°, 0°, 2.5°, 5°) and (− 15°, − 7.5°, 0°, 7.5°, 15°) were evaluated to investigate the effects from the angles for compounding the B-mode images.

**Fig. 3:**
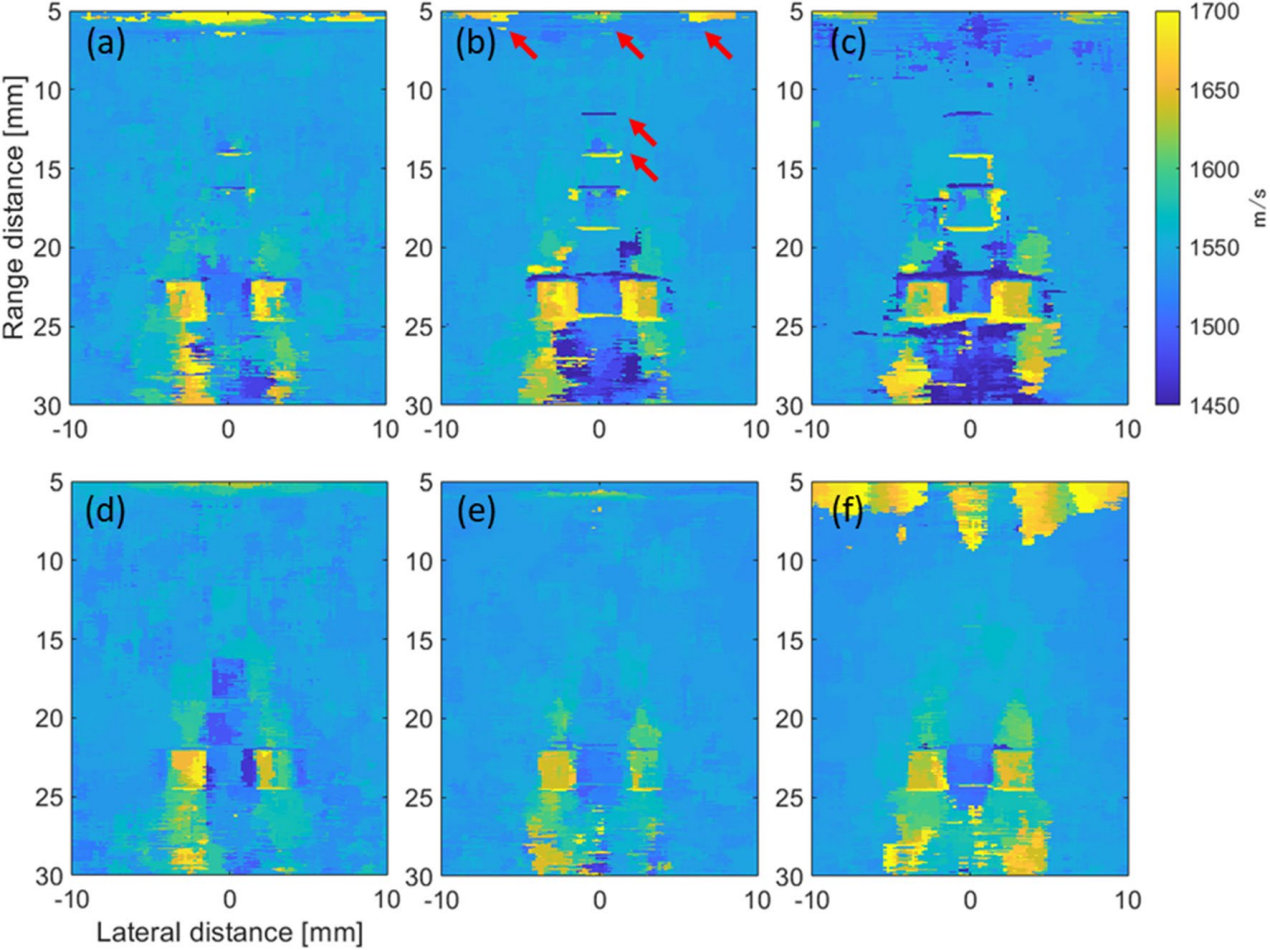
Average SoS distributions estimated from the CF obtained by **a** maximum estimation angle: 5 degrees, **b** maximum transmission angle: 10 degrees, and **c** maximum transmission angle: 15 degrees. Average SoS distributions estimated from the reciprocal of the phase variance obtained by **d** maximum transmission angle: 5 degrees, **e** maximum transmission angle: 10 degrees, and **f** maximum transmission angle: 15 degrees

**Fig. 4:**
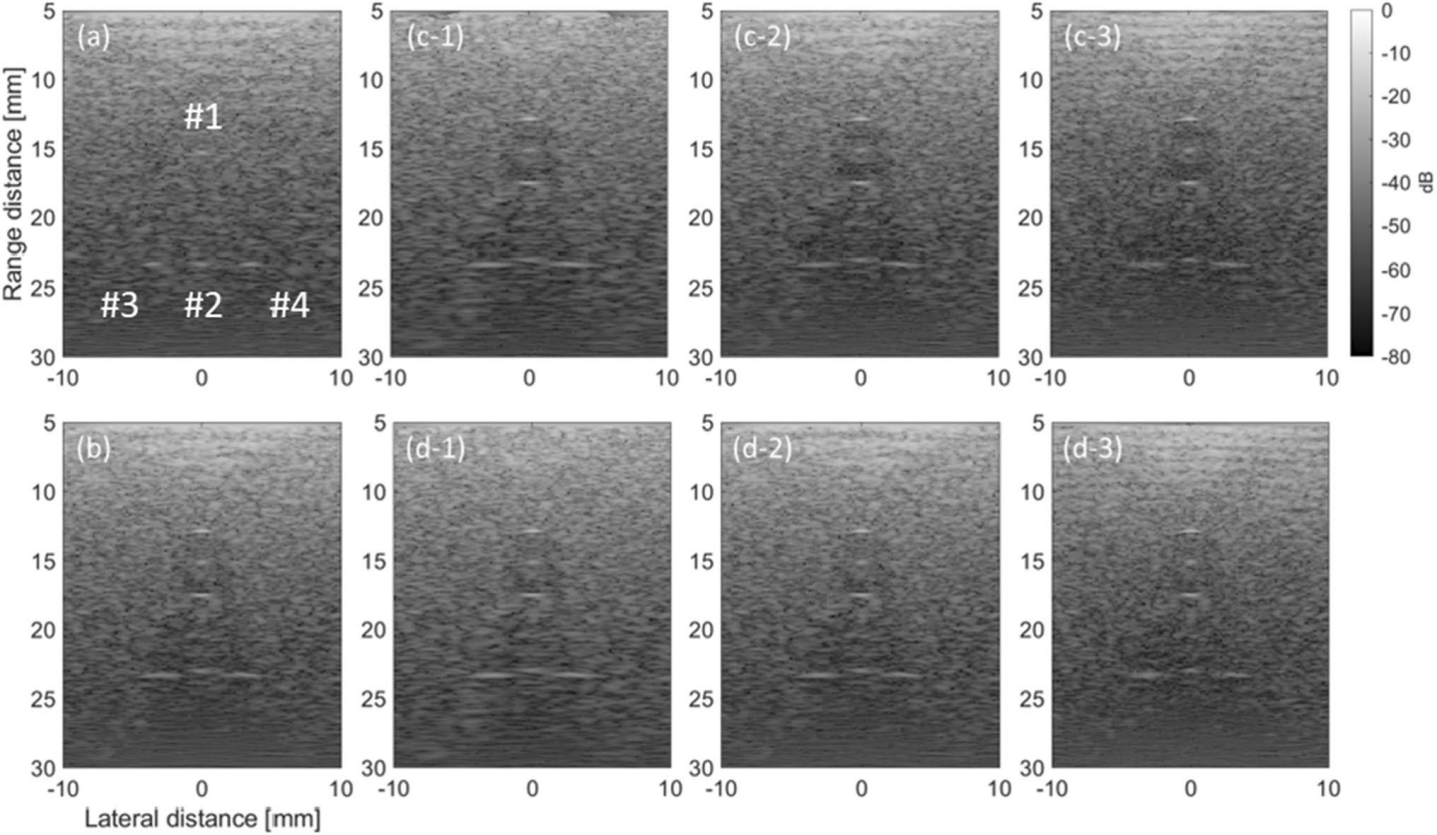
B-mode images of the phantoms **a** without and **b** with the cyst region reconstructed by assuming a constant average SoS of 1540 m/s at a maximum compound angle of 10 degrees, respectively. B-mode images of the phantom with the cyst reconstructed using average SoS distributions estimated from the CF (maximum estimation angle: 10 degrees) at maximum compound angles of **c-1** 5, **c-2** 10, and **c-3** 15 degrees. B-mode images of the phantom with the cyst reconstructed using average SoS distributions estimated from the reciprocal of phase variance (maximum estimation angle: 10 degrees) at maximum compound angles of **d-1** 5, **d-2** 10, and **d-3** 15 degrees

**Fig. 5:**
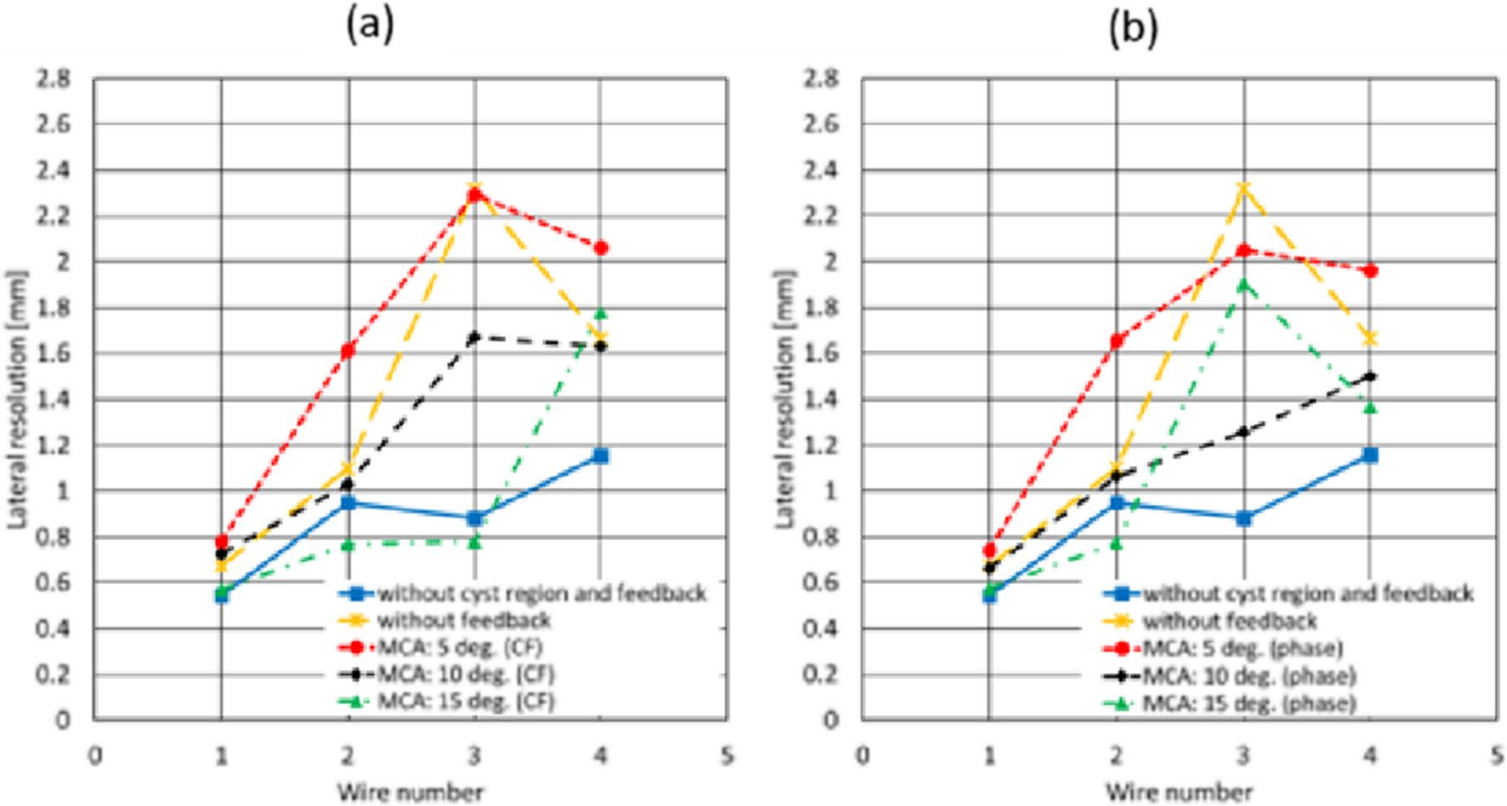
Comparisons of lateral resolutions when using average SoS distributions estimated from the **a** CF and **b** reciprocal of the phase variance for image reconstruction. MCA: maximum compound angle

Figure 3(a)–(c) shows the distributions of the average SoS estimated from the CF obtained using the maximum transmission angles of 5, 10, and 15 degrees, respectively. Figure 3(d)–(e) shows the distributions of the average SoS estimated from the reciprocal of the phase variance obtained using the maximum transmission angles of 5, 10, and 15 degrees, respectively. Note that while the distribution shown in Fig. 1 shows the assigned local SoS, the distributions in Fig. 3 show the average SoS. The estimates in Fig. 3(a)–(c) on the surface of the phantom and at the boundaries between the background and cyst regions, e.g., the regions indicated by the red arrows in Fig. 3(b), seemed to vary qualitatively. Meanwhile, the estimates in Fig. 3(d) and (e) seemed to be estimated as a uniform value compared to those in Fig. 3(a)–(c). Figure 4(a) and (b) shows the B-mode images of the phantoms without and with the cyst region reconstructed by assuming a constant average SoS of 1540 m/s at a maximum compound angle of 10 degrees, respectively. Figure 4(c-1)–(c-3) shows the B-mode images of the phantom with the cyst reconstructed using the average SoS distributions estimated from the CF (maximum estimation angle: 10 degrees) at the maximum compound angles of 5, 10, and 15 degrees, respectively. Figure 4(d-1)–(d-3) shows the B-mode images of the phantom with the cyst reconstructed using average SoS distributions estimated from the reciprocal of the phase variance (maximum estimation angle: 10 degrees) at the maximum compound angles of 5, 10, and 15 degrees, respectively. Then, the lateral resolutions were calculated from the envelope profiles of Fig. 4 in the lateral direction, and the calculated results are summarized in Fig. 5(a) and (b). Note that MCA is the abbreviation of the maximum compound angle. Figure 5(a) and (b) shows a comparison of the lateral resolutions without the feedback to those with the feedback using the average SoS estimated based on the CF and reciprocal of the phase variance, respectively. Compared to the results of the phantom without the cyst and feedback, the lateral resolutions of the wires in the cyst phantom ($\# 1$) and located in the deeper region ($\# 2$) without the feedback were slightly worse. Also, the lateral resolutions of the wires ($\# 3$ and $\# 4$) located in the deeper region were degraded. Meanwhile, the lateral resolutions with the feedback using estimates based on the CF and reciprocal were improved by focusing on the wires ($\# 3$ and $\# 4$) at a maximum compound angle of 10 degrees. For example, the lateral resolutions of the wire ($\# 3$) at a maximum compound angle of 10 degrees were 0.88, 2.32, 1.67, and 1.26 mm (w/o cyst region and feedback, w/ cyst and w/o feedback, w/ cyst and feedback (CF), and w/ cyst and feedback (phase)). Consequently, the lateral resolution obtained using the reciprocal was slightly improved compared with that obtained using the CF. It’s considered that although the diameter of the wire was small, as the assigned SoS of the wire was higher than those in the surrounding region, and there was a difference in acoustic impedances between wire and surrounding region, the boundary might have an influence on estimation of the average SoS. Although the lateral resolutions at a maximum compound angle of 15 degrees were basically improved in comparison with those at a maximum compound angle of 10 degrees, the signals from the surface and at the boundaries more influenced the estimated results, as shown in Fig. 3(c) and (f). Hence, the transmission sequence with steering angles of (− 10°, − 5°, 0°, 5°, 10°) was chosen for both estimation and compounding in the following in vivo experiments.

### In vivo experiments

**Fig. 6:**
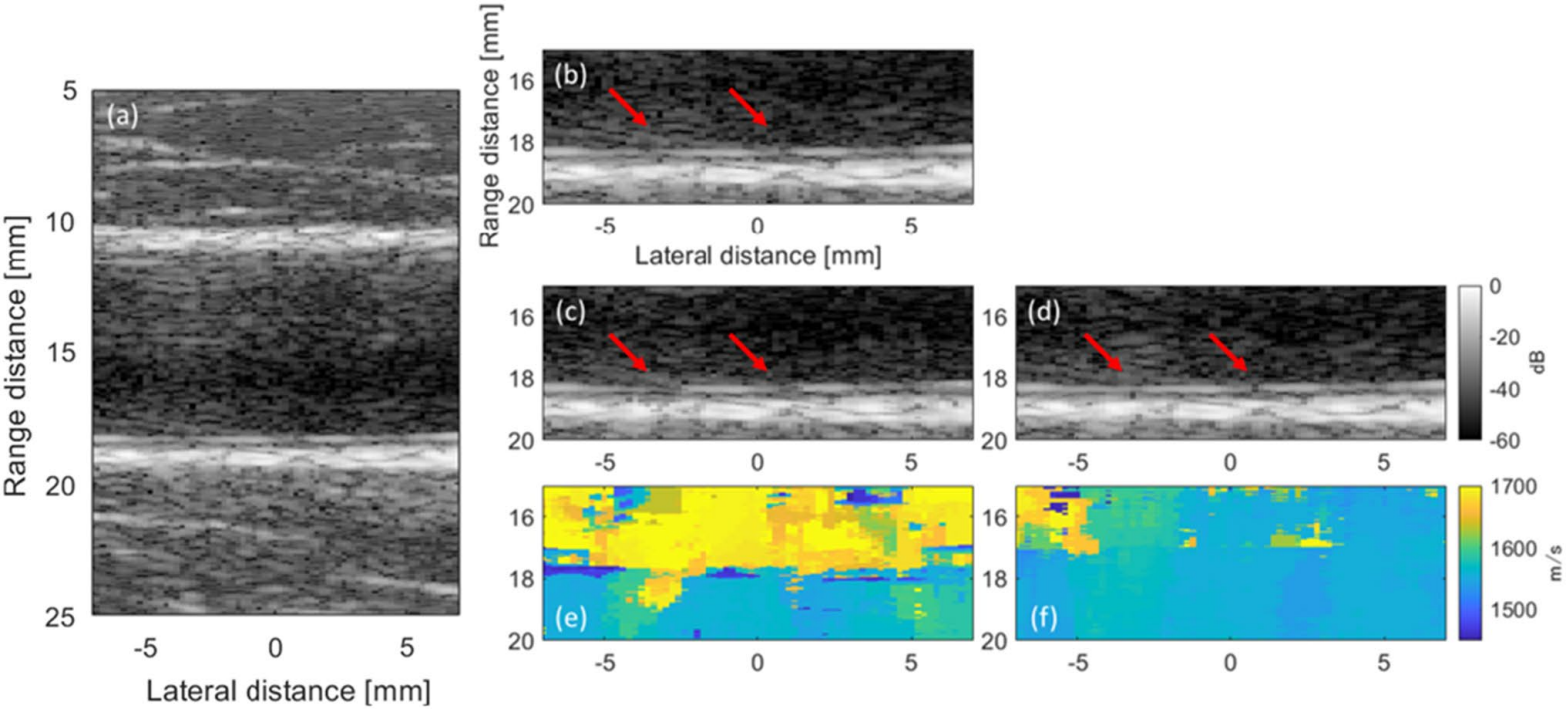
**a** B-mode image of the carotid artery reconstructed by assuming a constant average SoS of 1540 m/s. **b** Enlarged B-mode image of posterior wall of the carotid artery (Fig. 6(a)) reconstructed by assuming a constant average SoS of 1540 m/s. Enlarged B-mode images of posterior wall of the carotid artery reconstructed using average SoS distributions estimated from the **b** CF and **c** reciprocal of the phase variance (maximum estimation angle: 10 degrees). Estimated distributions based on the **e** CF and **f** reciprocal of the phase variance

Figure 6(a) shows a B-mode image of the carotid artery reconstructed by assuming a constant average SoS of 1540 m/s (w/o feedback). Also, Fig. 6(b) shows an enlarged B-mode image of the posterior wall of the carotid artery (Fig. 6(a)) reconstructed by assuming a constant average SoS of 1540 m/s (w/o feedback). Figure 6(c) and (d) shows enlarged B-mode images of the posterior wall of the carotid artery reconstructed using the average SoS distributions estimated from the CF (Fig. 6(e)) and reciprocal of the phase variance (Fig. 6(f)), respectively. Comparing Figs. 6(b)–(d), the continuity of the intima in the lateral direction was improved (indicated by arrows) by the feedback using the distribution estimated from the reciprocal of the phase variance in Fig. 6(d). Meanwhile, discontinuity of the intima was observed in Fig. 6(c) compared to the results without the feedback (Fig. 6(b)).

## Discussion

### Comparison of CF and reciprocal of phase variance at boundaries

**Fig. 7:**
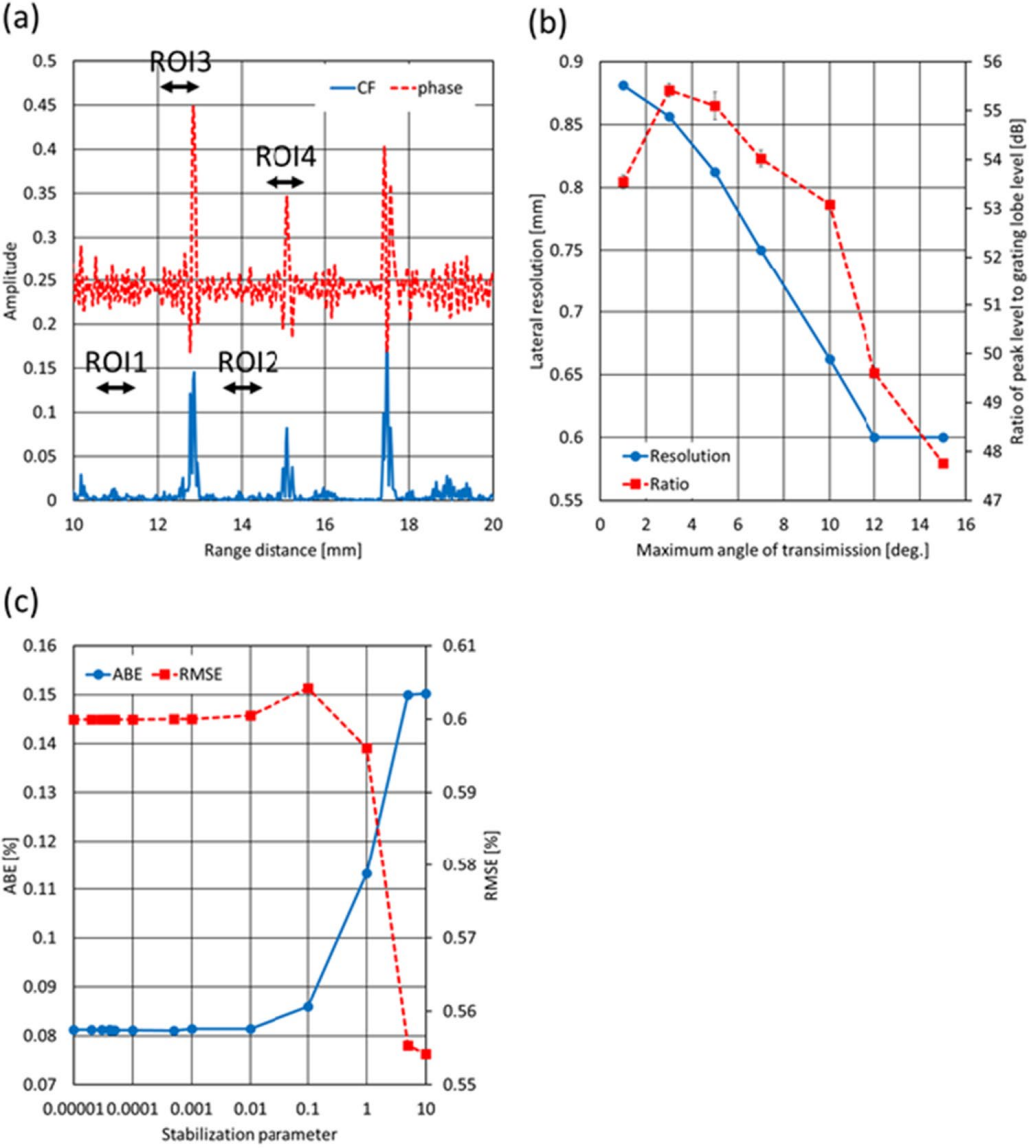
**a** CF and the reciprocal of phase variance values from 10 to 20 mm in the range direction at the center of Fig. 4(b). **b** Lateral resolution and ratio of peak level to grating lobe level under different maximum angles in plane transmissions for estimation. **c** Relationship between the stabilization parameter and estimation error

Figure 7(a) shows the CF and reciprocal of the phase variance values from 10 to 20 mm in the range direction at the center of Fig. 4(b). These values were calculated by assuming the average SoS as 1540 m/s. The average values of the CF not including the boundaries (ROI1: 10.5–11.5 mm, ROI2: 13.5–14.5 mm) were calculated to be 0.0064 and 0.005, respectively. The average values of the reciprocal not including the boundaries (ROI1: 10.5–11.5 mm, ROI2: 13.5–14.5 mm) were 0.25 and 0.25, respectively. Meanwhile, the average values of the CF including the boundaries (ROI3: 12–13 mm, ROI4: 14.5–15.5 mm) were 0.086 and 0.038, respectively. The average values of the reciprocal including the boundaries (ROI3: 12–13 mm, ROI4: 14.5–15.5 mm) were 0.32 and 0.27, respectively. From the calculated CF values, the ratios of the average value with the boundary to one without the boundary were 22.5 (ROI3 to ROI1) and 17.6 dB (ROI4 to ROI2), respectively. Meanwhile, the ratios regarding the calculated reciprocal values were 2.1 (ROI3 to ROI1) and 0.69 dB (ROI4 to ROI2), respectively. Hence, the ratios of the reciprocal were much smaller than those of the CF, and it’s considered that the estimation method using the reciprocal of the phase variance was less subject to the effects of the signals from the boundary.

### Effects of lateral resolution and grating lobe level on estimation accuracy

As shown in Fig. 2, the evaluation indices for estimation of the average SoS, i.e., ABE and RMSE, were the most suppressed at a maximum transmission angle of 10 degrees. We will discuss the estimation accuracy from the viewpoint of the lateral resolution and grating lobe level by performing a numerical simulation with a one-point target. The simulation conditions were identical to the ones described above. For the one-point target, one scatterer was located at a depth of 20 mm from the probe surface. In this simulation, the full width at half-maximum, i.e., the lateral resolution, of envelope profiles in the lateral direction at a depth where a maximum value was observed at the center of the scatterer was evaluated under different maximum transmission angles. Also, the ratio of the maximum value at the center of the scatterer (called the peak level) to the grating lobe level at the same depth was calculated. Figure 7(b) shows the lateral resolutions and ratios of the peak level to the grating lobe level under the different maximum angles in the plane wave transmissions, respectively. The lateral resolution reached the minimum at a maximum angle of 12 degrees and the constant value from this angle to a maximum angle of 15 degrees. Meanwhile, the ratio of the peak level to the grating lobe level was decreased by approximately 3 dB from a maximum angle of 10 degrees to 12 degrees. These results indicated that the estimation accuracy was improved by achieving a higher lateral resolution with increases of maximum transmission angles (1 to 10 degrees) and degraded by increases of the effects from the grating lobe (10 to 15 degrees).

### Effects of stabilization parameter on estimation accuracy

In this section, we will investigate the effects of the stabilization parameter λ in Eq. (8) on the estimation accuracy. One of the datasets obtained in the numerical simulations using the inhomogeneous phantom was employed for this investigation. Also, the maximum transmission angle for the estimation was set to 10 degrees. Figure 7(c) shows the ABE and RMSE plotted as a function of the stabilization parameter. In Fig. 7(c), the circle and squared dots correspond to the ABE and RMSE of the estimates, respectively. As shown in Fig. 7(c), the fluctuations of both the ABE and RMSE were small and within 0.1% at most. Hence, it’s considered that the values of the stabilization parameter have less effects on the estimation accuracy.

### Limitations

In this section, we discuss two limitations of the method for estimating the average SoS. The first limitation is related to the assumption for the estimation. In the estimation of the average SoS, in a case where the signals are not scattered from a point scatterer described in Sect. “Estimation method for average speed of sound”, there is a possibility that the estimate does not conform to the actual average SoS. In other words, in cases where the signals are scattered from a scatterer with a diameter of more than the wavelength of the transmitted waves, i.e., small vessels or a planer structure such as the arterial wall [9], the estimate could not conform to the actual average SoS. However, as the estimate corresponding to the average SoS when the evaluation index obtained using the delay-compensated element signals is maximized at an interest point in the receive beamforming, the image reconstruction using the estimate could improve the quality of the image such as lateral resolution. Hence, in the numerical simulation using the cyst phantom, the estimated distribution of the average SoS may not correspond with the assigned distribution due to the reasons described above and refraction. Also, the distributions of the average SoS in Fig. 6(e) and (f) might differ with the actual ones.

The second limitation is related to the propagation path of the transmitted plane waves. Although the evaluation index, i.e., CF or reciprocal of the phase variances, was averaged among the different transmission angles, the average SoS was dependent on the propagation path. When the region includes complex structures with different SoSs, the estimated average SoS in the region might be different with the actual one. For this reason, a method for estimating the average SoS using only the evaluation index at a specific angle or the average of those at some angles close to the specific angle will be investigated in a future study. However, there are several issues relating to lower SNR, directivity, and spatial resolution to achieve the proposed strategy.

## Conclusion

In this study, we investigated the accuracy of estimation of the average SoS based on the reciprocal of the phase variance compared to that based on the CF. For this purpose, two numerical simulations with the k-Wave software were performed. In the numerical simulation using an inhomogeneous phantom, the relationship between the estimation accuracy and transmission angles for the estimation was investigated, and the RMSEs obtained based on the CF and reciprocal of the phase variance were 1.25 ± 0.093 and 0.7645 ± 0.167% at the transmission sequence of steering angles of (− 10°, − 5°, 0°, 5°, 10°), respectively. Next, the effects of the signals from the boundaries and degree of improvement of the image quality using the estimates of the average SoS for image reconstruction were evaluated by performing numerical simulations using a cyst phantom containing wire targets. As a result, at a maximum compound angle of 10 degrees, the lateral resolutions with feedback using the CF and the reciprocal were improved compared to the results without the feedback, respectively. Finally, the estimation methods based on the CF and reciprocal were applied to in vivo data from the common carotid artery, and B-mode images were reconstructed using the estimated distributions of the average SoS. In a B-mode image reconstructed with the feedback using the estimates from the reciprocal, improvement of the continuity of the intima in the lateral direction was observed. Consequently, the results of both the numerical simulations and in vivo experiments indicated that the usage of the reciprocal of the phase variance at a maximum angle of 10 degrees was proper for estimation and compounding.

As shown in Figs. 6(e) and (f), the estimates in the lumen region of the carotid artery were different from those in the tissue region, and the effects from signals from flowing red blood cells in the lumen might lead to this difference. In future work, we will compare the distribution of the average SoS estimated when removing the signals from the red blood cells using a low-pass filter in the frame direction with the results obtained in this study. Also, in a future study, we will investigate the effects on estimation of the local SoS of the phantom and tissues using the average SoS based on the reciprocal under the assumption that the measurement target consists of layered structures (proposed in the referenced paper [31]). Also, in previous studies [27, 28], we proposed an estimation method using focused ultrasonic beams and revealed that the number of active transducer elements used for forming the ultrasonic beam had effects on the estimated results. Therefore, we will investigate the effects from 2D and 3D acoustic fields of the ultrasonic transmission, i.e., focused beam, plane wave, an acoustic lens, and the presence of the lens by performing numerical simulations and real measurements.

## Data Availability

The datasets analysed in this study are available from the corresponding author on reasonable request.
