# Correction: Investigation of a method to estimate the average speed of sound using phase variances of element signals for ultrasound compound imaging

**DOI:** 10.1007/s10396-024-01442-y

**Published:** 2024-04-03

**Authors:** Ryo Nagaoka, Masaaki Omura, Hideyuki Hasegawa

**Affiliations:** Faculty of Engineering, University of Toyama, 3190 Gofuku, Toyama, 930-8555 Japan

**Correction: Journal of Medical Ultrasonics (2023) 51:17–28** 10.1007/s10396-023-01378-9

The article “Investigation of a method to estimate the average speed of sound using phase variances of element signals for ultrasound compound imaging”, written by Ryo Nagaoka, Masaaki Omura and Hideyuki Hasegawa, was originally published Online First without Open Access. After publication in volume 51, issue 1, pages 17–28 the author decided to opt for Open Choice and to make the article an Open Access publication. Therefore, the copyright of the article has been changed to © The Author(s) 2023 and the article is forthwith distributed under the terms of the Creative Commons Attribution 4.0 International License, which permits use, sharing, adaptation, distribution and reproduction in any medium or format, as long as you give appropriate credit to the original author(s) and the source, provide a link to the Creative Commons licence, and indicate if changes were made. The images or other third party material in this article are included in the article’s Creative Commons licence, unless indicated otherwise in a credit line to the material. If material is not included in the article’s Creative Commons licence and your intended use is not permitted by statutory regulation or exceeds the permitted use, you will need to obtain permission directly from the copyright holder. To view a copy of this licence, visit http://creativecommons.org/licenses/by/4.0.

The original article has been corrected.

